# Herbs-Partitioned Moxibustion Combined with Acupuncture Inhibits TGF-*β*1-Smad-Snail-Induced Intestinal Epithelial Mesenchymal Transition in Crohn's Disease Model Rats

**DOI:** 10.1155/2019/8320250

**Published:** 2019-05-30

**Authors:** Yin Shi, Tao Li, Jing Zhou, Yuwei Li, Liu Chen, Haixia Shang, Yajing Guo, Yi Sun, Jimeng Zhao, Chunhui Bao, Huangan Wu

**Affiliations:** Shanghai Institute of Acupuncture Moxibustion and Meridian, Shanghai University of Traditional Chinese Medicine, Shanghai 200030, China

## Abstract

Crohn's disease may cause excessive damage and repair in the intestinal epithelium due to its chronic relapsing intestinal inflammation. These factors may initiate the TGF-*β* 1-Smad pathway to activate the transcription factor of Snail, and the Snail-mediated pathway promotes the transformation of intestinal epithelial cells to mesenchymal cells, leading to intestinal fibrosis. Acupuncture and moxibustion have been demonstrated to prevent intestinal fibrosis in Crohn's disease. However, it is not clear whether acupuncture and moxibustion can inhibit intestinal epithelial mesenchymal transformation in Crohn's disease by affecting the TGF-*β* 1-Smad-Snail pathway. This study indicated that abnormal increased expressions of TGF*β*1, T*β*R2, Smad3, and Snail were significantly downregulated by herbs-partitioned moxibustion at Tianshu (ST25) and Qihai (RN6) and acupuncture at Zusanli (ST36) and Shangjuxu (ST37). In addition, protein and mRNA levels of E-cadherin, the epithelial cell marker, were significantly increased. Protein and mRNA levels of fibronectin, the mesenchymal cell marker, were decreased in the intestinal tissue. Moreover, the number of mesenchymal cells in the intestinal mucosa can be reversely transformed to intestinal epithelial cells. Therefore, herbs-partitioned moxibustion combined with acupuncture can prevent intestinal epithelial mesenchymal transition by inhibiting abnormal expression of TGF*β*1, T*β*R2, Smad3, and Snail in the TGF-*β*1-Smad-Snail pathway in Crohn's disease.

## 1. Introduction

Crohn's disease (CD) is a chronic inflammatory disease in the digestive tract that can affect any parts of the digestive system, ranging from the mouth to the anus. At present, the etiology of CD is still unclear, and the clinical prognosis includes a long duration and frequent recurrence [1]. One characteristic symptom of CD is intestinal fibrosis, which results in stenosis. Repeated inflammation of the intestine caused by excessive damage repair of CD was reported to induce intestinal epithelial mesenchymal transition (EMT), resulting in intestinal stenosis [2]. Approximately 1/3 of CD patients underwent surgeries due to intestinal strictures or obstructions; however, after surgery, 70% of patients suffered from intestinal obstruction [3, 4]. The recurrence of intestinal inflammation and stenosis reduces CD patients' quality of life.

Although the mechanism of intestinal inflammation in CD has been studied clearly, however, intestinal fibrosis and stenosis has been rarely investigated. Studies on the onset of intestinal fibrosis in recent years have been demonstrated that EMT is an essential cause leading to intestinal fibrosis in CD, while TGF-*β*1-Smad-Snail pathway is one of the major pathways of EMT occurrence [5]. Previous studies have shown that acupuncture and moxibustion have effects in preventing intestinal fibrosis of CD [6–12]. In this study, CD rats with trinitrobenzene sulphonic acid (TNBS) were applied to discuss effect of TGF-*β*1-Smad-Snail in inducing intestinal EMT of CD. Meanwhile, the role of herbs-partitioned moxibustion combined with acupuncture in preventing intestinal EMT of CD through TGF-*β*1-Smad-Snail pathway was studied as well.

## 2. Results

### 2.1. The Morphological Changes of Intestinal Epithelial Tissues in Different Groups

**Figure 1:**
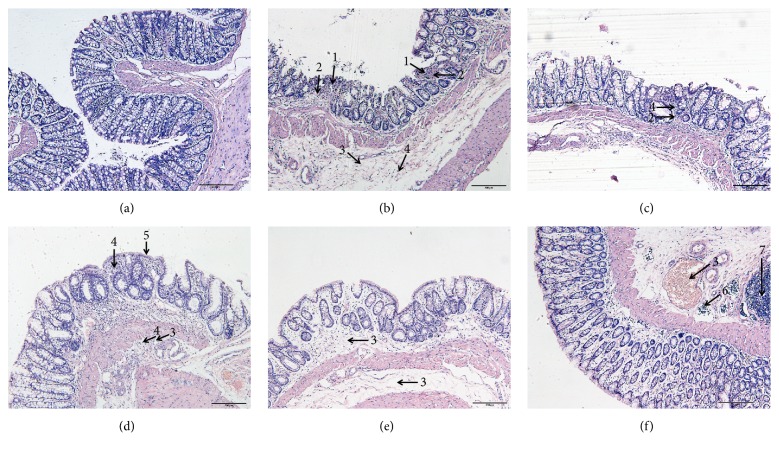
*Representative histological observation from a light microscope of the rat colonic epithelial tissue sections stained with HE (magnification×100)*. (a) Normal control; (b) model control; (c) salicylazosulfapyridine; (d) acupuncture; (e) herbs-partitioned moxibustion; (f) herbs-partitioned moxibustion combined with acupuncture. Arrowheads: (1) multiple superficial and small ulcers; (2) fibrous tissue; (3) congestion and edema; (4) inflammatory cells; (5) epithelial proliferation and repair; (6) lymphatic dilatation; (7) intestinal wall of lymphatic tissue proliferation.

In the NC group, intact mucosal epithelial cells and muscularis and regular gland arrangement were observed, and no abnormal changes were observed in the submucosa (Figure 1(a)).

In the MC group, multiple small and shallow ulcers were found in the mucosal epithelia, and no ulcers were found in the muscularis. Necrotic tissue exudation was found on the surface of the ulcer, and proliferation of the fibrous tissue was found under the ulcer. In addition, hyperaemia, edema, and infiltration of inflammatory cells were observed under the submucosa (Figure 1(b)).

In the SASP group, damage and abscission occurred in a small amount of mucous epithelial cells. The size of the mucous glands was unequal, and the arrangement was in disorder. Infiltration of a small number of inflammatory cells and proliferation of fibrous tissue were observed around the glands (Figure 1(c)).

In the AC group, hyperplasia and repair were observed in the local mucosal epithelium. The mucosal gland was of different sizes and shapes, and mild proliferation occurred in the glandular epithelium. Inflammatory cells infiltrated the interstitium with no fibrous hyperplasia. Mild hyperaemia, edema, and a small number of inflammatory cells were observed under the submucosa (Figure 1(d)).

In the HPM group, hyperaemia and edema were observed in the mucosal epithelium with the infiltration of a few inflammatory cells. Hyperaemia and edema were observed under the submucosa as well (Figure 1(e)).

In the HPM+AC group, lymphatic dilatation, vascular dilatation, congestion, and edema were observed only under the submucosa. In addition, infiltration of a small number of inflammatory cells and proliferation of the intestinal lymphoid tissue were observed (Figure 1(f)).

### 2.2. The Expression of TGF*β*1, T*β*R1, T*β*R2, Smad3, and Snail in the Intestinal Epithelial Tissues of Different Groups

**Figure 2:**
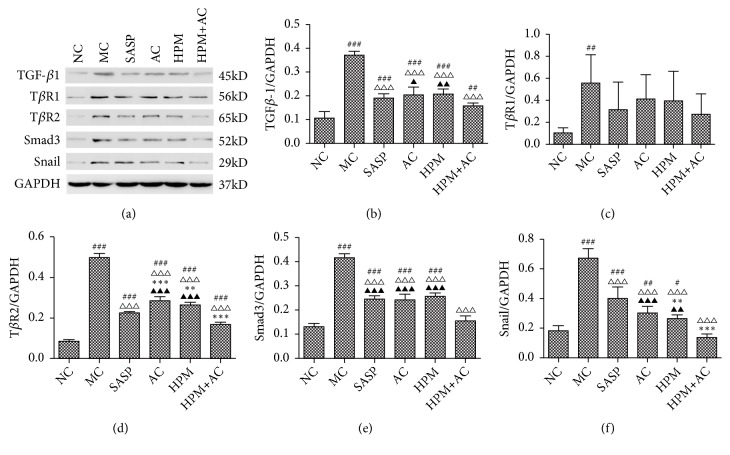
*The protein expression levels of TGF-β1, TβR1, TβR2, Smad3, and Snail and the corresponding GAPDH were determined using Western blot analysis*. The following histogram shows the quantitative densitometry of the blots in each group (n= 10 for each group). White space was used to make explicit for the grouping of blots cropped from different parts of the same gel or from different gels. Values are the means ± SD. In all panels, ###*P*<0.001, ##*P*<0.01, and #*P*<0.05 vs. NC. △△△*P*<0.001 vs. MC. ▲▲▲*P* <0.001, ▲▲*P* <0.01, and ▲*P *<0.05 vs. HPM+AC.

Compared to the NC group, the expression levels of TGF-*β*1 in the intestinal epithelial tissues in the MC, SASP, AC, HPM, and HPM+AC groups were significantly increased (*P*MC, SASP, AC, HPM <0.001, and* P*HPM+AC<0.01). Compared to the MC group, there was a significant decrease in the expression level of TGF-*β*1 in the SASP, AC, HPM, and HPM+AC groups (*P*all<0.001). The expression level of TGF-*β*1 in the HPM+AC group was lower than that in the AC and HPM groups (*P*=0.01;* P*<0.01) (Figures 2(a) and 2(b)).

Compared to the NC group, the expression level of T*β*R1 in the intestinal epithelial tissues in the MC group was markedly increased (*P*<0.01), while those of the SASP, AC, HPM, and HPM+AC groups showed no significant differences (*P*all>0.05). Compared to the MC group, there were no significant differences in the expression levels of T*β*R1 in the SASP, AC, HPM, and HPM+AC groups (*P*all>0.05) (Figures 2(a) and 2(c)).

Compared to the NC group, the expression levels of T*β*R2 in the intestinal epithelial tissues in the MC, SASP, AC, HPM, and HPM+AC groups were significantly increased (*P*all<0.001). Compared to the MC group, there was a significant decrease in the expression level of T*β*R2 in the SASP, AC, HPM, and HPM+AC groups (*P*all<0.001). However, compared to the SASP group, the expression levels of T*β*R2 in the AC and HPM groups were significantly increased (*P*<0.001;* P*=0.001), and the expression level in the HPM+AC group was significantly decreased (*P*<0.001). The expression level of T*β*R2 in the HPM+AC group was lower than that in the AC and HPM groups (*P*all<0.001) (Figures 2(a) and 2(d)).

Compared to the NC group, the expression levels of Smad3 in the intestinal epithelial tissues in the MC, SASP, AC, and HPM groups were significantly increased (*P*all<0.001), while no significant difference in the HPM+AC group was found (*P*>0.05). Compared to the MC group, there was a significant decrease in the expression level of Smad3 in the SASP, AC, HPM, and HPM+AC groups (*P*all<0.001). The expression level of Smad3 in the HPM+AC group was lower than that in the SASP, AC, and HPM groups (*P*all<0.001) (Figures 2(a) and 2(e)).

Compared to the NC group, the expression levels of Snail in the intestinal epithelial tissues in the MC, SASP, AC, and HPM groups were significantly increased (*P*MC, SASP<0.001,* P*AC<0.01, and* P*HPM<0.05), while no significant difference was observed in the HPM+AC group (P>0.05). Compared to the MC group, there was a significant decrease in the expression level of Snail in the SASP, AC, HPM, and HPM+AC groups (*P*all<0.001). The expression level of Snail in the SASP group was higher than that in the HPM+AC, HPM, and AC groups (*P*<0.001,* P*=0.001, and* P*<0.05), and in the HPM+AC group, it was lower than that in the AC and HPM groups (P<0.001; P<0.01) (Figures 2(a) and 2(f)).

### 2.3. The Expression of E-Cadherin and Fibronectin in the Intestinal Epithelial Tissues of Different Groups

**Figure 3:**
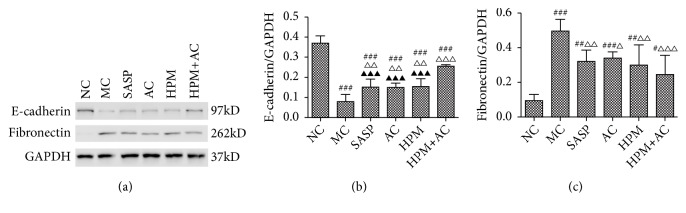
*The protein expression levels of E-cadherin and fibronectin and the corresponding GAPDH were determined using Western blot analysis*. The following histogram shows the quantitative densitometry of the blots in each group (n= 10 for each group). White space was used to make explicit for the grouping of blots cropped from different parts of the same gel or from different gels. Values are the means ± SD. In all panels, ###* P*<0.001, ##*P*<0.01, and #*P*<0.05 vs. NC. △△△*P*<0.001, △△*P*<0.01, and △*P*<0.05 vs. MC. ▲▲▲*P*<0.001 vs. HPM + AC.

Compared to the NC group, the expression levels of E-cadherin in intestinal epithelial tissues in the MC, SASP, AC, HPM, and HPM+AC groups were significantly increased (*P*all<0.001). Compared to the MC group, there was a significant decrease in the expression level of E-cadherin in the HPM+AC, SASP, AC, and HPM groups (*P*HPM+AC<0.001,* P*SASP, AC, and HPM <0.01). The expression level of E-cadherin in the HPM+AC group was higher than that in the SASP, AC, and HPM groups (*P*all<0.001) (Figures 3(a) and 3(b)).

Compared to the NC group, the expression levels of fibronectin in the intestinal epithelial tissues in the MC, SASP, AC, HPM, and HPM+AC groups were significantly increased (*P*MC<0.001,* P*SASP=0.001,* P*AC<0.001,* P*HPM<0.01, and* P*HPM+AC<0.05). Compared to the MC group, there was a different decrease in the expression level of fibronectin in the HPM+AC, SASP, HPM, and AC groups (*P*HPM+AC<0.001,* P*SASP, HPM<0.01, and* P*AC<0.05) (Figures 3(a) and 3(c)).

### 2.4. The Expression of E-Cadherin and Fibronectin mRNA in the Intestinal Epithelial Tissues of Different Groups

**Figure 4:**
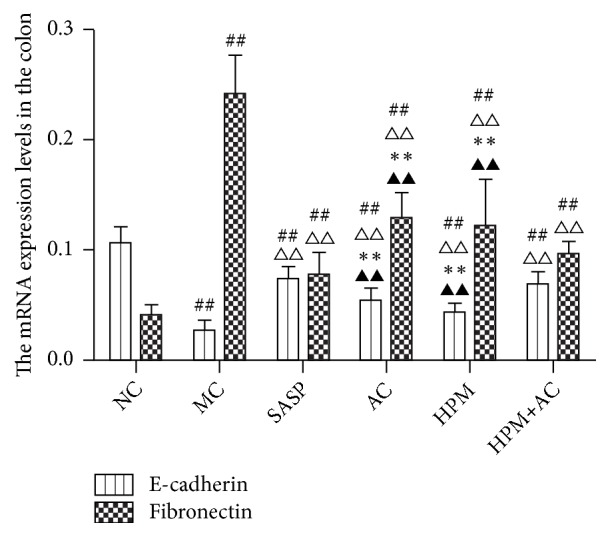
*Graphic representation shows the expression levels of E-cadherin mRNA and fibronectin mRNA analyzed by quantitative RT-PCR* (n=10, for each group). Values are the means ± SD. ##*P*<0.01 vs. NC. △△*P* <0.01 vs. MC. *∗∗P *<0.01 vs. SASP. ▲▲*P *<0.01 vs. HPM+AC.

Compared to the NC group, the expression levels of E-cadherin mRNA in the intestinal epithelial tissues in the MC, SASP, AC, HPM, and HPM+AC groups were significantly decreased (*P*all<0.01). Compared to the MC group, there was a significant increase in the expression level of E-cadherin mRNA in the SASP, AC, HPM, and HPM+AC groups (*P*all<0.01). Compared to the SASP and HPM+AC groups, the expression levels of E-cadherin mRNA in the AC and HPM groups were markedly decreased (*P*all<0.01) (Figure 4).

Compared to the NC group, the expression levels of fibronectin mRNA in intestinal epithelial tissues in the MC, SASP, AC, HPM, and HPM+AC groups were significantly increased (*P*all<0.01). Compared to the MC group, there was a significant decrease in the expression level of fibronectin mRNA in the SASP, AC, HPM, and HPM+AC groups (*P*all<0.01). Compared to the SASP and HPM+AC groups, the expression levels of fibronectin mRNA in the AC and HPM groups were markedly increased (*P*all<0.01) (Figure 4).

### 2.5. The Coexpression of Snail/E-Cadherin and Snail/Fibronectin in the Intestinal Epithelial Tissues of Different Groups

Compared to the NC group, the luminance value ratio of Snail in the intestinal epithelial tissues in the MC, SASP, AC, HPM, and HPM+AC groups was significantly increased (*P*all<0.001), the luminance value ratio of E-cadherin was significantly decreased (*P*MC, AC, HPM<0.001, and* P*SASP =0.001), and there was no significant difference in the HPM+AC group (*P*>0.05). Compared to the MC group, the luminance value ratio of Snail in the SASP, AC, HPM, and HPM+AC groups was significantly decreased (*P*all<0.001), and the luminance value ratio of E-cadherin in the HPM+AC, SASP, HPM, and AC groups was markedly increased (*P*HPM+AC<0.001,* P*SASP=0.001,* P*HPM<0.01, and* P*AC<0.05). Meanwhile, the luminance value ratio of Snail was lower than that in the HPM, AC, and SASP groups (*P*HPM=0.001,* P*AC<0.01, and* P*SASP<0.05), and the luminance value ratio of E-cadherin was higher than that in the AC and HPM groups (*P*AC<0.01;* P*HPM<0.05) in the HMP+AC group.

**Figure 5:**
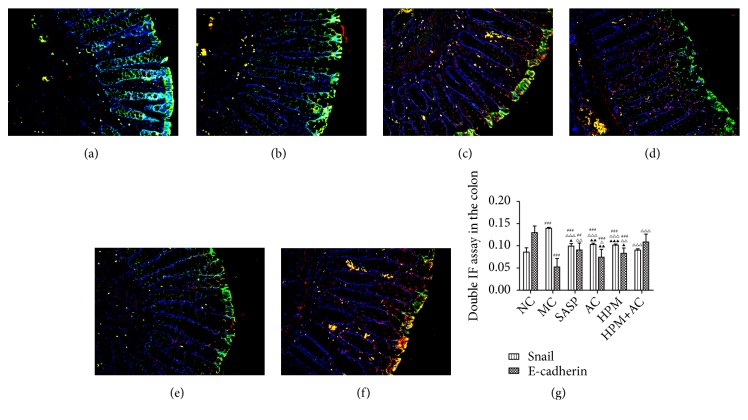
*Coexpression of Snail and E-cadherin in the rat intestinal epithelial tissues from the NC, MC, SASP, AC, HPM, and HPM+AC groups* (n=10, for each group). (a) Normal control; model control; (c) salicylazosulfapyridine; (d) acupuncture; (e) herbs-partitioned moxibustion; (f) herbs-partitioned moxibustion combined with acupuncture. (g) The histogram shows the coexpression of Snail and E-cadherin in each group (n= 10, for each group). Values are the means ± SD. In all panels, ###* P*<0.001 and ##*P*<0.01 vs. NC. △△△*P*<0.001, △△*P*<0.01, and △*P*<0.05 vs. MC. ▲▲*P* <0.01 and ▲*P* <0.05 vs. HPM+AC. Scale bar ×200. Labelled for Snail (red), E-cadherin (green), Snail, and E-cadherin coexpression (yellow).

In Figures 5(a)–5(f), the nuclei are presented as blue fluorescent, Snail expression is red fluorescent, and E-cadherin expression is green fluorescent, while Snail and E-cadherin coexpression are yellow fluorescent mixed with red fluorescent and green fluorescent. The immunofluorescence images present some instances of red fluorescent Snail expression, green fluorescent E-cadherin expression and yellow fluorescent Snail and E-cadherin coexpression in the NC group (Figure 5(a)). There are many instances of red fluorescent Snail expression, and a few instances yellow fluorescent Snail and E-cadherin coexpression are displayed in the MC group (Figure 5(b)). More instances of yellow fluorescent Snail and E-cadherin coexpression and a few of red fluorescent Snail expression are shown in the SASP (Figure 5(c)), AC (Figure 5(d)), and HPM groups (Figure 5(e)). Many instances of green fluorescent E-cadherin expression and a few of yellow fluorescent Snail and E-cadherin coexpression are displayed in the HPM+AC group (Figure 5(f)).

Compared to the NC group, the luminance value ratio of Snail in the intestinal epithelial tissues in the MC, AC, SASP, and HPM groups was significantly increased (*P*MC<0.001,* P*AC<0.01,* P*SASP, and HPM<0.05), and there was no significant difference in the HPM+AC group (*P*>0.05), while the luminance value ratio off fibronectin in the intestinal epithelial tissues in the MC, AC, SASP, HPM, and HPM+AC groups was significantly increased (*P*MC, SASP, AC, HPM<0.001, and* P*HPM+AC<0.05). Compared to the MC group, the luminance value ratio of Snail in the HPM+AC, SASP, AC, and HPM groups was markedly decreased (*P*HPM+AC<0.001,* P*SASP, AC, and HPM<0.05), and the luminance value ratio of fibronectin in the HPM+AC, HPM, SASP, and AC groups was markedly decreased (*P*HPM+AC<0.001,*P*HPM<0.01,* P*SASP, and AC<0.05). Meanwhile, the luminance value ratio of Snail was lower than that in the SASP, AC, and HPM groups (*P*all<0.05), and the luminance value ratio of fibronectin was lower than that in the SASP and AC groups (*P*both<0.05).

**Figure 6:**
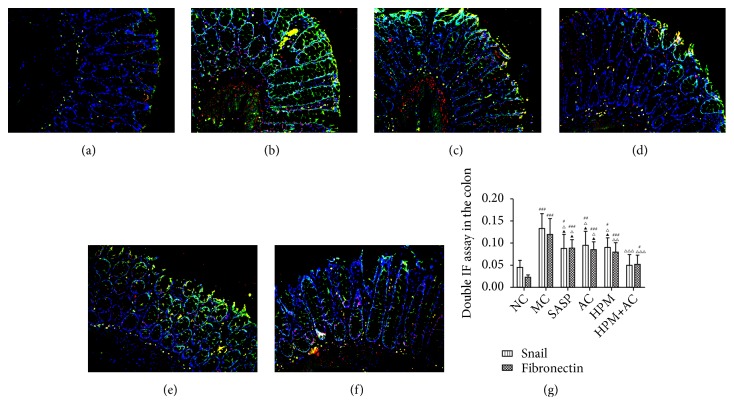
*Coexpression of Snail and fibronectin in the rat intestinal epithelial tissues from the NC, MC, SASP, AC, HPM, and HPM+AC groups* (n=10 for each group). (a) Normal control; model control; (c) salicylazosulfapyridine; (d) acupuncture; (e) herbs-partitioned moxibustion; (f) herbs-partitioned moxibustion combined with acupuncture. (g) The histogram shows the coexpression of Snail and E-cadherin in each group (n= 10, for each group). Values are the means ± SD. In all panels, ###*P*<0.001, ##*P*<0.01, and #*P*<0.05 vs. NC. △△△*P*<0.001, △△*P*<0.01, and △*P*<0.05 vs. MC. ▲*P*<0.05 vs. HPM+AC. Scale bar ×200. Labelled for Snail (red), fibronectin (green), Snail, and fibronectin coexpression (yellow).

In Figures 6(a)–6(f), the nuclei were presented as blue fluorescent, Snail expression was red fluorescent, fibronectin expression was green fluorescent, and Snail and fibronectin coexpression were yellow fluorescent mixed with red fluorescent and green fluorescent. The immunofluorescence images displayed a few instances of red fluorescent Snail expression and green fluorescent fibronectin expression and a trace of yellow fluorescent Snail and fibronectin coexpression in NC group (Figure 6(a)). Many instances of green fluorescent fibronectin expression were shown, alternating with yellow fluorescent Snail and fibronectin coexpression in MC group (Figure 6(b)). A few instances of yellow fluorescent Snail and fibronectin coexpression patterns were displayed in the SASP (Figure 6(c)), AC (Figure 6(d)), and HPM groups (Figure 6(e)). A trace of yellow fluorescent Snail and fibronectin coexpression was shown in the HPM+AC group (Figure 6(f)).

## 3. Discussion

The present study indicates that mechanism of CD related to EMT is via the activation of TGF-*β*1-Smad-Snail pathway. In addition, herbs-partitioned moxibustion combined with acupuncture was the most effective method to inhibit EMT by decreasing levels of TGF-*β*1, Smad3, and Snail in TGF-*β*1-Smad-Snail pathway.

Cryptogenic abscess, inflammatory cells of the submucosal tissue, and formation of granulomas are found in CD [13, 14]. Chronic inflammation and tissue repair of the intestinal tract lead to fibrosis-like features, such as stenosis of the intestinal cavity and changes in intestinal wall compliance [15]. During onset of CD-induced intestinal fibrosis, EMT was shown to be an essential pathway [16]. Our present study showed absent goblet cells and expanded glands with irregular shapes and variable sizes and massive fibrous tissues in the mucosa and submucosa in CD model rats. A successful CD model establishment is showed. For the CD treatment, SASP has demonstrated a routine medication in CD [17]. In addition, acupuncture (AC) and herbs-partitioned moxibustion (HPM) have been demonstrated to be efficient treatment methods in CD [18–20]. Therefore, in this study, we established SASP group as a standard treatment method to compare to AC, HPM, and HPM+AC group to find out the best treatment method for CD. The morphological observation showed epithelia and glands in alignment with no fibrosis, slight edema in the submucosa, hyperaemia in lymphatic vessels, a few fibrous tissues, and closely joined enterocytes in HPM+AC group which had a better improvement than other treatment groups. The result suggested that herbs-partitioned moxibustion combined acupuncture can treat ulcer, erosion, fibrosis, and edema in CD intestine.

TGF-*β*1 is the main driving force of EMT and promotes tissue fibrosis [21]. One mechanism of EMT is overexpression of TGF-*β*1 binding to T*β*R1 and T*β*R2, thus activating the protein expression of Smad3 and Snail in the downstream pathway [22, 23]. In this study, the MC group and all the treatment groups showed significantly higher expression levels of TGF-*β*1 in the intestinal epithelial tissues than that of the NC group. Expression levels of TGF-*β*1 in all the treatment groups were markedly lower than that in the MC group, especially in HPM+AC group. As for T*β*R1 and T*β*R2, both of them increased in MC group compared to NC group. However, only expression of T*β*R2 in HPM+AC group had significant difference compared to other treatment groups. The results indicate that HPM+AC group is more effective than other treatment groups in reducing TGF-*β*1 and T*β*R2 expressions in the intestinal epithelial tissues of CD rat models.

Smad3, a signaling transduction intermediate downstream of TGF-*β*1 and activin receptors, conducting the signals of TGF-*β*1 into the nucleus, is essential for TGF-*β*1-induced EMT [24]. Both increased expressions of TGF-*β*1 and Smad3 have been demonstrated in TNBS colitis [25]. In the present study, expression level of Smad3 in MC group was much higher than that in NC group. Expression levels of Smad3 in all treatment groups were decreased remarkably compared to that in MC group, particularly in HPM+AC group which was similar to that in NC group. In addition, we found that expression levels of Smad3 and TGF-*β*1 were showed the same tendency in MC group and all treatment groups. Herbs-partitioned moxibustion combined with acupuncture is the best treatment to downregulate expression of Smad3, and TGF-*β*1 can induce the expression of its downstream protein through Smad3 protein.

Snail is a major transcription factor inducing EMT. Upregulated expression of Snail has been indicated in the process of EMT [26]. In our study, both protein and mRNA levels of Snail in all treatment groups showed a significant decrease compared to those in MC group. Expression levels of Snail in HPM+AC and SASP groups were lower than those in AC and HPM groups, especially in HPM+AC group. Results showed that herbs-partitioned moxibustion combined with acupuncture are the most effective way to reduce the expression of Snail. In addition, the expression level of Snail changes along with TGF-*β*1, suggesting that Snail is controlled by the TGF-*β*1. Taken together, reduced expression of TGF-*β*1 decreases Snail and avoids occurrence of EMT.

In the process of EMT, Snail is a strong repressor of E-cadherin gene transcription [27]. Olmeda et al. demonstrated that silencing of Snail can upregulate E-cadherin and downregulate fibronectin in the process of EMT [28]. Protein and mRNA levels of E-cadherin in MC group had a significant decrease compared to those in all other groups in the present study, while protein and mRNA levels of fibronectin had a marked increase compared to those in all other groups. Protein and mRNA levels of E-cadherin in HPM+AC group showed a higher increase than those in other treatment groups. In addition, immunofluorescence images showed that significant increased expressions of Snail and fibronectin and marked decreased expressions of E-cadherin in MC group compared to those in NC group. In all treatment groups, the expression levels of Snail and fibronectin were significant decreased while expression level of E-cadherin was marked increased compared to those in MC group. Among the treatment groups, HPM+AC group was the most effective treatment in regulating Snail, fibronectin and E-cadherin. All the results indicate that herbs-partition moxibustion combined with acupuncture could regulate the abnormal expression of E-cadherin and fibronectin by reducing Snail expression, thus further preventing intestinal EMT in CD induced by the TGF-*β*1-Smad-Snail pathway.

In conclusion, herbs-partition moxibustion combined with acupuncture could inhibit the overexpression of TGF-*β*1, T*β*R2, Smad3, and Snail in the TGF-*β*1-Smad-Snail pathway, upregulate protein and mRNA levels of E-cadherin, and downregulate protein and mRNA levels of fibronectin, thus preventing intestinal EMT in CD.

## 4. Materials and Methods

### 4.1. Experimental Animals

Experiments were carried out on Sprague-Dawley (SD) rats (male, SPF, 140 ± 10 g) purchased from the Shanghai University of Traditional Chinese Medicine. After arrival, the rats were on a 12 hr light-dark cycle with sufficient water and food provided. All the procedures were approved by the ethics committee of Shanghai University of Traditional Chinese Medicine (no. 2015013) and were conducted in strict accordance with the recommendations of the Guidelines for the Care and Use of Shanghai Laboratory Animal Center. The number of animals used along with their suffering was minimized.

### 4.2. Groups and CD Model Establishment

Rats were subdivided into the following 6 groups: the normal control group (NC), the model control group (MC), the SASP control group (SASP), the acupuncture control group (AC), the herbs-partitioned moxibustion control group (HPM), and the herbs-partitioned moxibustion combined with acupuncture control group (HPM+AC). Based on our previous experience, with the assumption that Herbs-partition Moxibustion and Acupuncture took more effect than the model control group, equal to or more than eight rats in each group were needed to yield a power of 80% with a significance level of 0.025 (2pair-wise comparisons). Thus, the sample sizes were ten rats in each group.

Prior to model establishment, all rats were provided only water for 24 h. With the exception of the normal control group that was injected with saline, rats in all the other groups received TNBS/ethanol (100 mg/kg TNBS+50% ethanol, 0.25 ml) through the anus by a rubber tube; the solution was retained in the gut cavity at a depth of 6-8 cm. Rats were fixed in a handstand posture for 1 min after the rubber tube was drawn away to prevent solution overflow. This procedure was performed on days 7, 14, 21, 28, 35, and 42 [29, 30]. No rat models died during model establishment.

### 4.3. Herbs-Partition Moxibustion and Acupuncture Treatment

HPM+AC group (n=10): in the HPM+AC group, moxa cones (0.5 cm in diameter, 0.3 cm in length, 90 mg in weight) made of refined mugwort floss were ignited after being placed on an herbal cake that was made of yellow rice wine and powdered herbs (Radix Aconiti Lateralis Preparata, Cortex Cinnamomi, Rhizoma Coptidis, Radix Aucklandiae, Radix Salviae Miltiorrhizae, Flos Carthami, etc.) at Tianshu (ST25, bilateral) and Qihai (RN6). Two moxa cones were applied at each acupoint in a single treatment once daily for 14 days. After local disinfection, disposable sterile acupuncture needles (0.25×25 mm, Hwato, Suzhou Medical Appliance Factory) were punctured perpendicularly 2-3 mm in depth at Zusanli (ST36, bilateral) and Shangjuxu (ST37, bilateral) and remained in place for 10 min during a single treatment. The rats in the HPM+AC group received both treatments once daily for 14 days. The acupoints were located according to the Experimental Acupuncture Science edited by Li Zhongren (China Press of Traditional Chinese Medicine, 2007).

HPM group (n=10): in the HPM group, Tianshu (ST25, bilateral) and Qihai (RN6) were selected for moxibustion. The treatment was the same as the moxibustion in the HPM+AC group.

AC group (n=10): in the AC group, Zusanli (ST36, bilateral) and Shangjuxu (ST37, bilateral) were punctured. The treatment was the same as the acupuncture in the AC group.

SASP group (n=10): in the SASP group, according to the Experimental Methodology of Pharmacology by Xu Shuyun (Beijing: People's Medical Publishing House), mesalazine solution (Dr. Falk Pharma, Germany; Lot No. 01B06515LA) at a proportion of 0.018:1 was fed by lavage. The rats received lavage twice a day for 14 days. MC group (n=10): the rats in the MC group were fixed as the other rats were, but without any treatment. NC group (n=10): the rats in the NC group were fixed as the other rats were, but without any treatment.

### 4.4. Histological Observation

After treatments, all rats were sacrificed simultaneously. Approximately 6 cm of the colon lesion was reserved at 6-8 cm distance from the anus. A 1-cm length of the dissected colon was removed, washed with iced saline, fixed in 10 % formalin, embedded in paraffin, sectioned, stained with hematoxylin and eosin, dehydrated in 95, 90, and 70 % ethanol, cleared in xylene, mounted in Permount or Histoclad, and observed under a light microscope.

### 4.5. Western Blotting

Sixty micrograms of protein extracted from the isolated rat intestinal epithelial tissues samples was separated by SDS-PAGE and transferred to a PVDF membrane. Blots were blocked for 1 h in 5 % BSA and then incubated with primary antibodies for overnight at room temperature [4]. Primary antibodies were directed against TGF*β*1 (1:500) (Ab64715, Abcam), T*β*R1 (1:750) (Ab31013, Abcam), T*β*R2 (1:500) (Ab186838, Abcam), smad3 (1:1000) (#9523, CST), Snail (1:1000) (Ab180714, Abcam), E-cadherin (1:1000) (Ab76055, Abcam), and Fibronectin (1:1500) (Ab6328, Abcam). The films were visualized using an enhanced chemiluminescence system (Pierce Company, Minneapolis, MN, USA).

### 4.6. Real-Time PCR

Total cellular RNA isolated from the rat intestinal epithelial tissues samples using Trizol reagent 12*μ*l of total RNA was used as a template for reverse transcription using a superscript reverse transcriptase kit (Invitrogen, Carlsbad, CA, USA). The cDNA samples were then applied for PCR with the following primer pairs: for GAPDH, sense 5′ GTCGGTGTGAACGGATTTG 3′ and antisense 5′ TCCCATTCTCAGCCTTGAC 3′; for E-cadherin sense 5′ CCTCCTGCTCCTACTGTTTC 3′ and antisense 5′ TCTTCTCCACCTCCCTCTTC 3′; for Fibronectin sense 5′ GCTTAGGCCAAGACCATACC 3′ and antisense 5′ CTCTTCGTCAGTGCCAACAG 3′. Real-time QPCR was performed with a Quanti Tect SYBR green PCR kit (ABI, USA) using an ABI7500 real-time PCR system; mRNA expression data were calculated with the 2MMCt method normalized to the expression of GAPDH. The fold change of target gene cDNA relative to glyceraldehyde-3-phosphate dehydrogenase (GAPDH) endogenous control was determined as follows: FC=2-△△Ct, where△△Ct = (CtTarget-CtGAPDH) test - (CtTarget-CtGAPDH) control. Ct values were defined as the number of the PCR cycles at which the fluorescence signals were detected.

### 4.7. Double Immunofluorescent Labelling

Embedding tissue in paraffin, A cryostat (Reichert-Jung, Leica, Deerfield, IL, USA) was used to produce 5 *μ*m sections, which submerged in the xylene-substitute HemoDe for 30 minutes in order to deparaffinize the tissue section, rehydrated by 5-minute washes in graded alcohols (ethanol 100%, 95%, 80%, and 75%), and washed in distilled water. Mark the slides and let them dry for 30 min in the calorstat (65). Slides were submerged in the xylene-substitute HemoDe for 30 minutes in order to deparaffinize the tissue section. slides were submerged in a solution containing 0.01mM sodium citrate buffer solution, placed for 15 minutes, and then washed three times in 0.02M PBS for 3 minutes. The unconjugated primary antibodies targeting of E-cadherin (1:250) (ab76055, Abcam, Cambridge UK) or Fibronectin (1:100) (ab6328, Abcam, Cambridge UK) or Snail (1:200) (Ab180714, Abcam, Cambridge UK) which diluted in PBS were added to each tissue section and slides were placed in a moist chamber and incubated overnight at 4°C. The secondary antibody diluted in PBS was added to each tissue section and then washed three times in 0.02M PBS for 3 minutes. Slides were dried and cover slipped and then visualized using Nikon Eclipse fluorescent microscope equipped.

### 4.8. Statistical Analysis

The statistical software SPSS19.0 (Chicago, IL) was used for statistical analysis. All data were presented as mean± standard error of the mean (SEM). Statistics among each experimental group was analyzed using a Student t test or a one-way ANOVA followed by Bonferroni's post hoc test. In all analyses, statistical significance was considered at P< 0.05.

## Data Availability

The initial data used to support the findings of this study are included within the article.
